# Supplementary material for: Single-molecule imaging with cell-derived nanovesicles reveals early binding dynamics at a cyclic nucleotide-gated ion channel
Source: Nat Commun. 2021 Nov 9;12:6459. doi: 10.1038/s41467-021-26816-5 (PMC8578382; doi:10.1038/s41467-021-26816-5)
Supplement: Supplementary file 1 — Supplementary Information [file 41467_2021_26816_MOESM1_ESM.pdf]

Supplementary Material for "Single-molecule imaging with cell-derived nanovesicles reveals early binding dynamics at a cyclic nucleotide-gated ion channel"

Vishal R. Patel[1,2], Arturo M. Salinas[3], Darong Qi[1], Shipra Gupta[1], David J. Sidote[1], and Marcel P. Goldschen-Ohm[1]*

[1]Department of Neuroscience, The University of Texas at Austin
[2]Dell Medical School, The University of Texas at Austin
[3]Department of Physics, The University of Texas at Austin

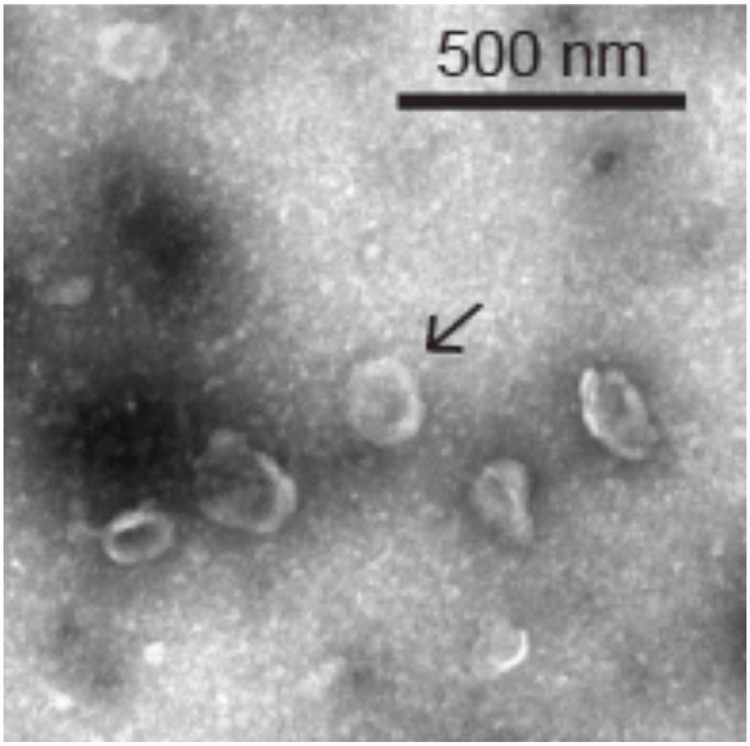

Supplementary Fig. 1 Transmission electron microscopy (TEM) images of cell-derived nanovesicles. Vesicle diameters in TEM images of a standard vesicle preparation (see Methods) ranged from approximately 50–1000 nm in diameter similar to previous reports based on dynamic light scattering[1]. Arrow indicates a single vesicle with a diameter of ~150 nm. Seven grid locations were imaged from a single vesicle preparation.

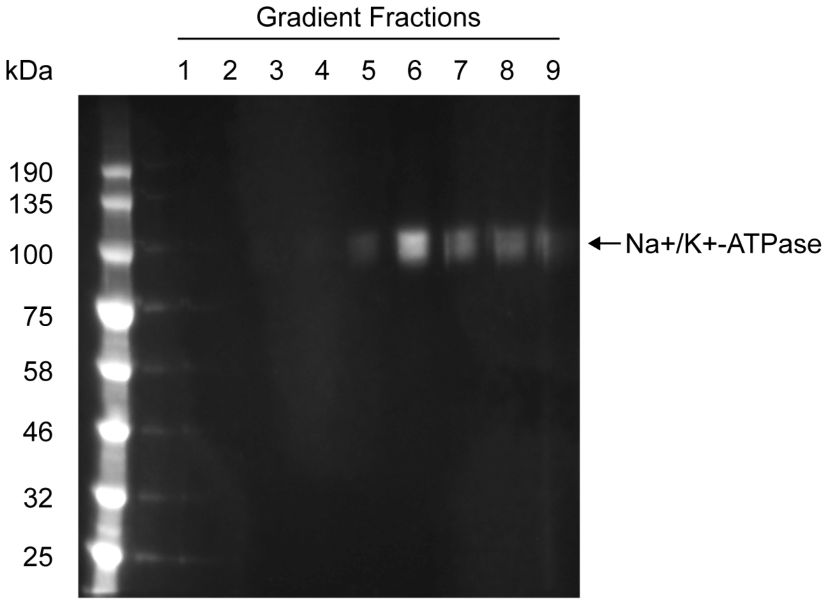

Supplementary Fig. 2 Western blot of plasma membrane fraction labeling. The left-most lane corresponds to molecular weight markers used as a standard. The same amount of nanovesicle lysate from fractions 1-8 following density gradient ultracentrifugation was loaded into the corresponding wells. Fractions containing nanovesicles derived from plasma membrane were detecting using fluorescent antibodies specific to Na+/K+-ATPase, which were enriched in plasma membrane fractions 5-8. Gel is for a single vesicle preparation, and results confirm an earlier report[2].

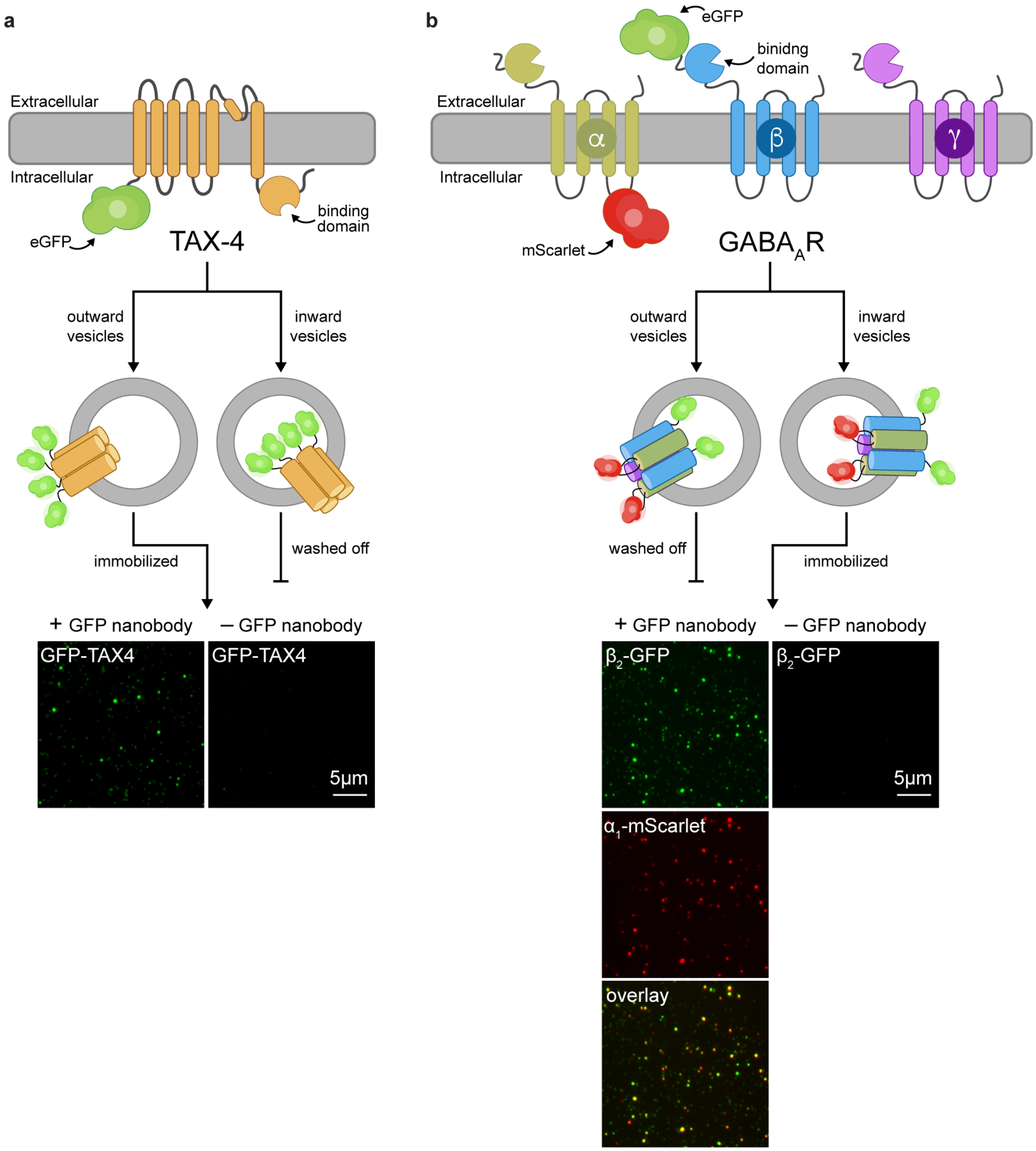

Supplementary Fig. 3 Specific immobilization of nanovesicles containing a membrane protein of interest. a Cartoon illustrating topology of a GFP-TAX-4 subunit with intracellular cyclic nucleotide binding domain and vesicles derived from cells expressing GFP-TAX-4 tetrameric channels. Below are mmTIRF images of GFP fluorescence (green) showing specific immobilization of GFP-TAX-4 containing vesicles at GFP-nanobodies deposited on the imaging surface (left) and a lack of appreciable nonspecific surface adsorption of vesicles in the absence of GFP-nanobodies (right). In both cases chambers were incubated in GFP-TAX-4 containing vesicles and then rinsed with buffer before imaging. **b** Cartoon illustrating topology of GABA$_A$ receptor α_1, β_2 and γ_{2L} subunits and vesicles derived from cells expressing heteropentameric

channels. For visualization and immobilization mScarlet was inserted in the intracellular M3-M4 linker of α_1 (α_1-mScarlet) and EGFP was inserted near the N-terminus of β_2 (β_2-GFP) on the same side of the membrane as the extracellular agonist and benzodiazepine binding domains. Below are mmTIRF images of GFP (green) and mScarlet (red) fluorescence showing colocalization (yellow) of β_2-GFP and α_1-mScarlet subunits at vesicles immobilized with GFP-nanobodies and a lack of appreciable nonspecific surface adsorption of vesicles in the absence of GFP-nanobodies.

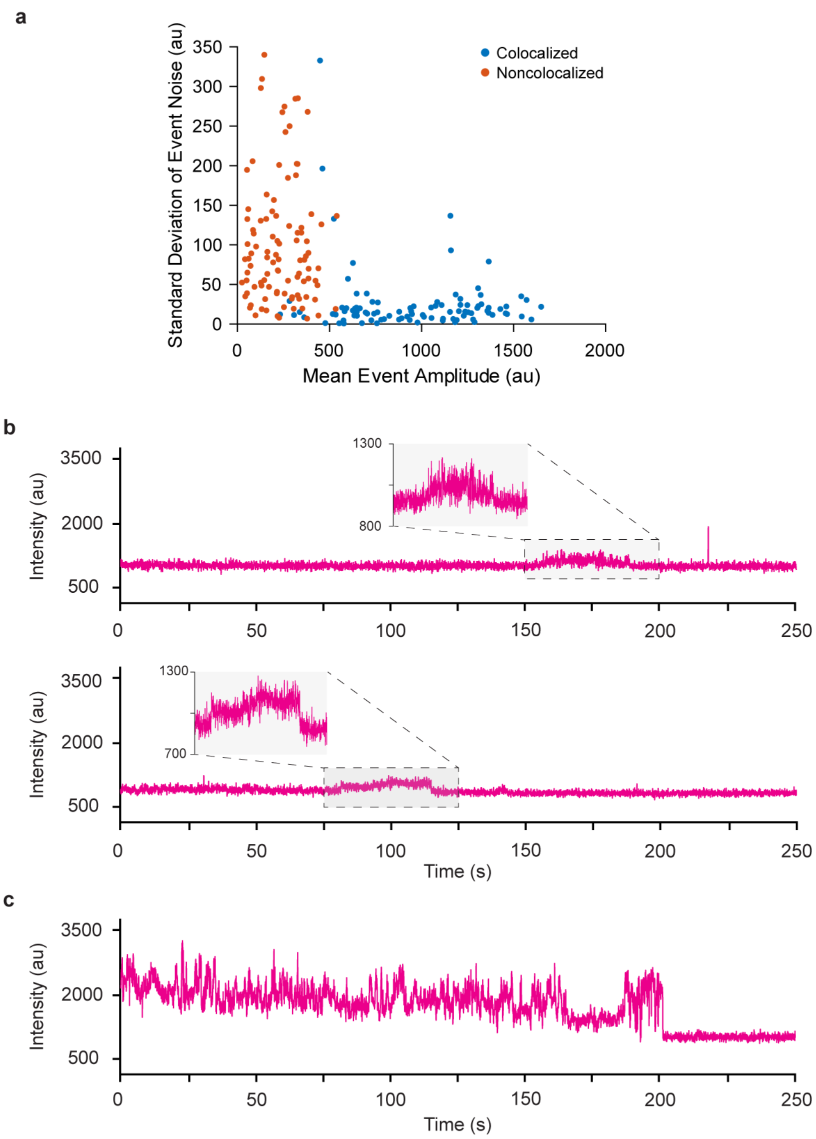

Supplementary Fig. 4 Distinct properties between colocalized and non-colocalized fcGMP binding events. a A comparison of the mean and standard deviation of the fluorescence signal from individual fcGMP binding events at spots that either do or do not colocalize with GFP-TAX-4 in a single field of view. The non-colocalized binding events are likely due to fluorescent contaminants in the PEG layer or non-specific adsorption of fcGMP to imperfections in the surface passivation. **b** Representative fluorescence intensity time series in arbitrary units (au) for low-intensity non-colocalized fcGMP signals, which make up a majority of the nonspecific signal. **c** Representative fluorescence intensity time series for high-intensity non-colocalized fcGMP signal likely due to non-specific adsorption to glass imaging surface. These high intensity traces make up a minority of observed non-colocalized signals.

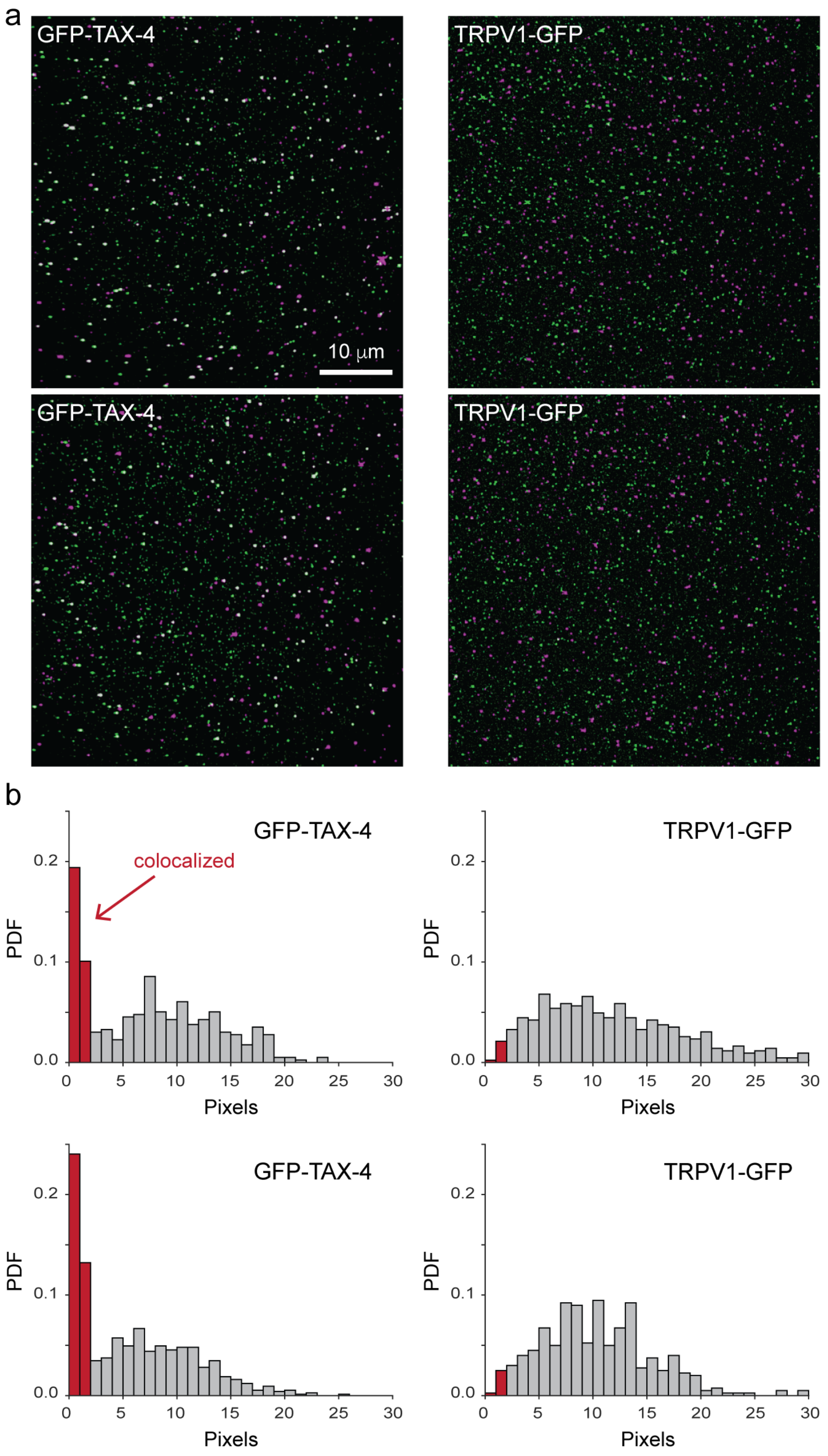

Supplementary Fig. 5 Summary of GFP and fcGMP colocalization for vesicles with GFP-TAX-4 and controls for vesicles with TRPV1-GFP channels. a Time averaged fluorescence for GFP (green) from nanovesicles containing either GFP-TAX-4 (left) or TRPV1 channels fused with an intracellular GFP (right) overlaid with 10 nM fcGMP (magenta) from the same field of view

(two separate imaging locations shown for each construct). Colocalized GFP and fcGMP signals appear white. Note the relative absence of colocalization for TRPV1 channels as compared to TAX-4 channels. Also note that some of the fainter green spots are subthreshold as only the brighter more distinct spots were observed in the presence but not absence of GFP. Results were similar for four separate experiments. **b** Histograms of the nearest neighbor distance from each identified GFP location to the closest identified fcGMP location. Two separate examples are shown for both GFP-TAX-4 and TRPV1-GFP vesicle preparations. The percentage (mean ± standard deviation) of GFP spots that colocalized with fcGMP spots (i.e., were within 3 pixels) across the dataset were for GFP-TAX-4 (at each fcGMP concentration): 33 ± 9% (10 nM), 32 ± 9% (30 nM), 31 ± 7% (60 nM), 33% (100 nM), and 39 ± 3% (200 nM). For TRPV1-GFP at 10 nM fcGMP a background colocalization of 7 ± 0.5% was observed.

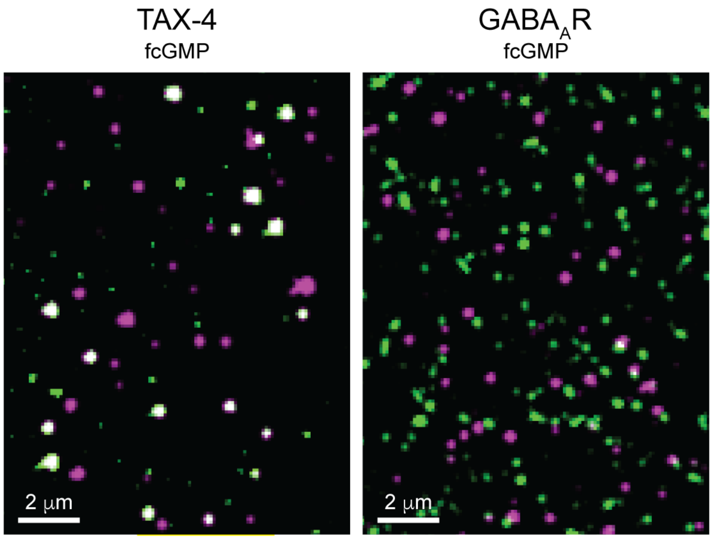

Supplementary Fig. 6 No colocalization between immobilized GABA$_A$R containing nanovesicles and fcGMP. Time averaged fluorescence for GFP (green) from nanovesicles containing either GFP-TAX-4 (left) or GABA$_A$ receptor α_1, β_2 and γ_{2L} subunits with GFP inserted in the N-terminus of the β_2 subunit (β_2-GFP) (right) overlaid with 100 nM fcGMP (magenta) from the same field of view. Colocalized GFP and fcGMP signals appear white. See Supplementary Fig. 3 for a description of the GABA$_A$R subunits. Results were similar for two separate experiments.

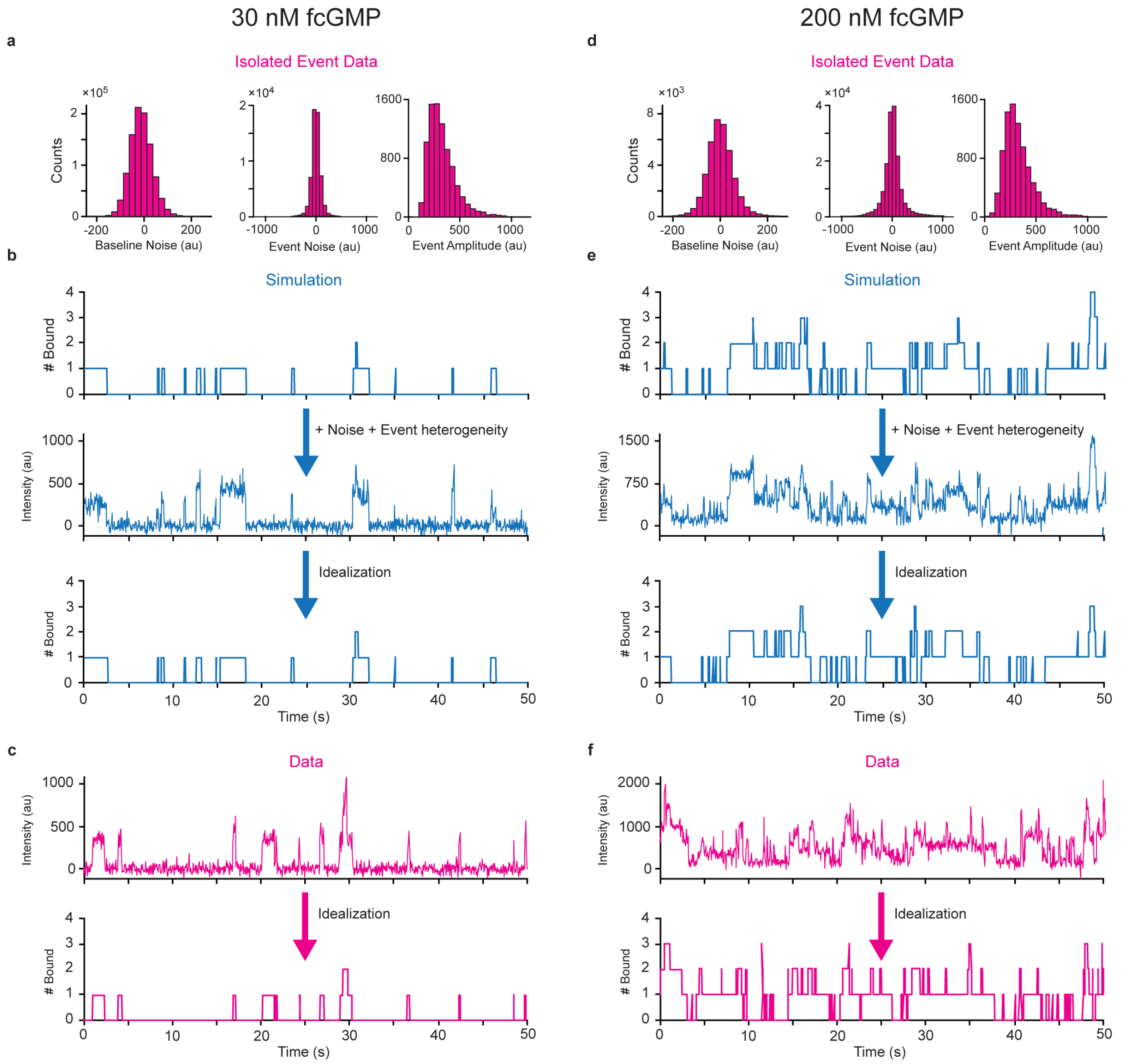

Supplementary Fig. 7 Evaluating the accuracy of idealization of fluorescence time series for ligand binding using simulated data. a Distributions of baseline noise, event noise, and event amplitudes for experimental binding data from single, isolated events at 30 nM fcGMP. **b** Example of time series from independent site binding simulations, followed by addition of gaussian noise and event amplitude heterogeneity representing isolated event data (a), followed by the results of our idealization procedure. Simulated intensities are in arbitrary units (au). Comparison of the known simulated time series to the results of our idealization procedure after adding noise drawn from experimental observations provides a metric for testing the accuracy of our idealization procedure (see Methods in main text). **c** Example of binding data at 30 nM fcGMP, followed by the results of our idealization procedure. **d-f** Same as described for **a-c**, but for simulations and data at 200 nM fcGMP.

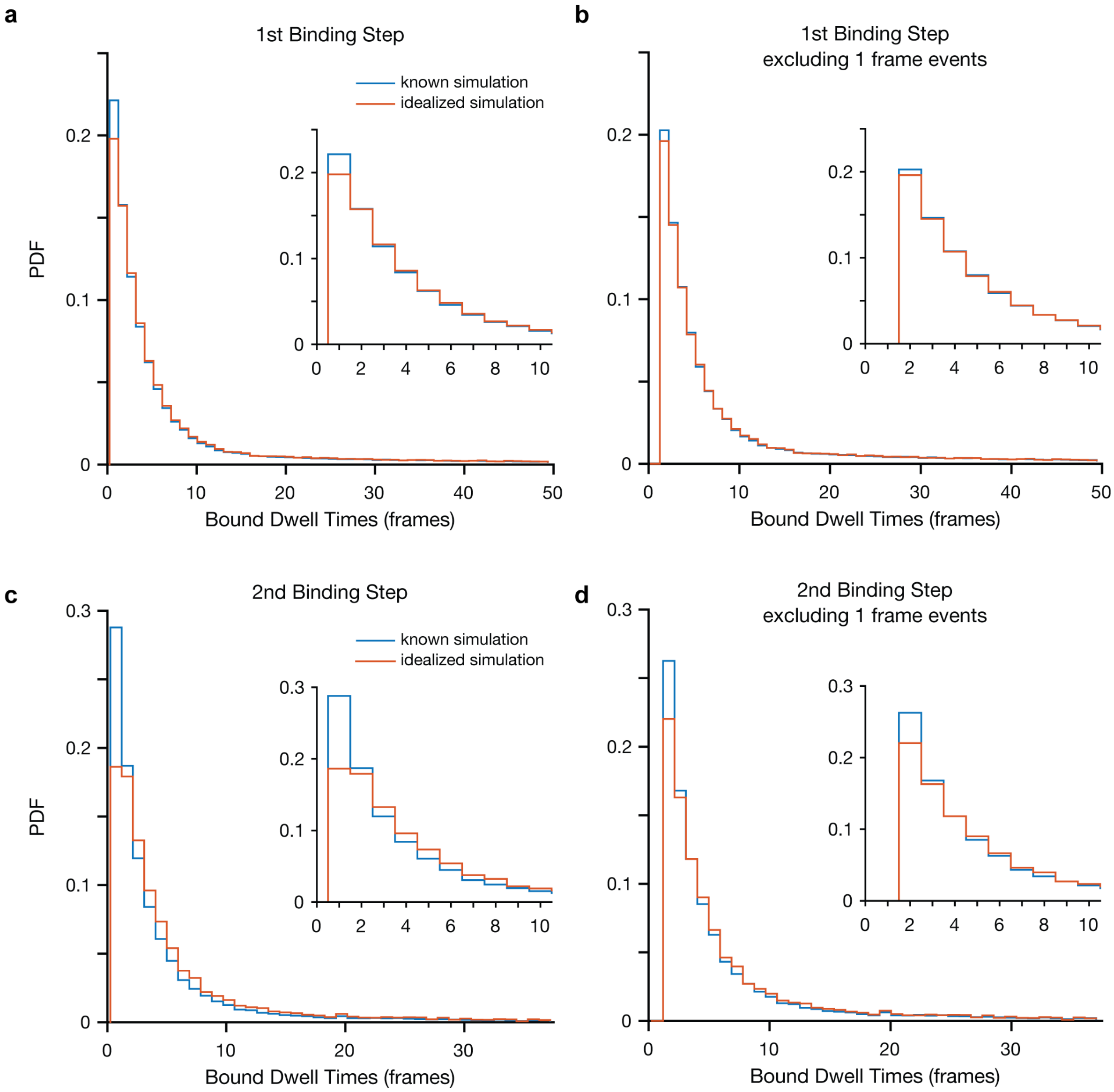

Supplementary Fig. 8 Missed events during idealization of simulated fluorescence time series. a-b Simulated bound dwell time distributions of isolated singly bound events either including (a) or excluding (b) single frame events. Note that durations are shown in frames. Comparison of known simulated bound series (blue) to the idealized bound series obtained from idealization after adding experimentally relevant noise and event heterogeneity (red). The primary discrepancy is limited to events lasting only a single frame, which are sometimes missed during the idealization procedure. However, nearly all events lasting two or more frames are reliably detected. **c-d** Same as described for **(**a-b**)**, but for dwell times in doubly bound states. All simulations were run with a frame rate of 20 Hz (50 ms per frame), equivalent to experimental recordings.

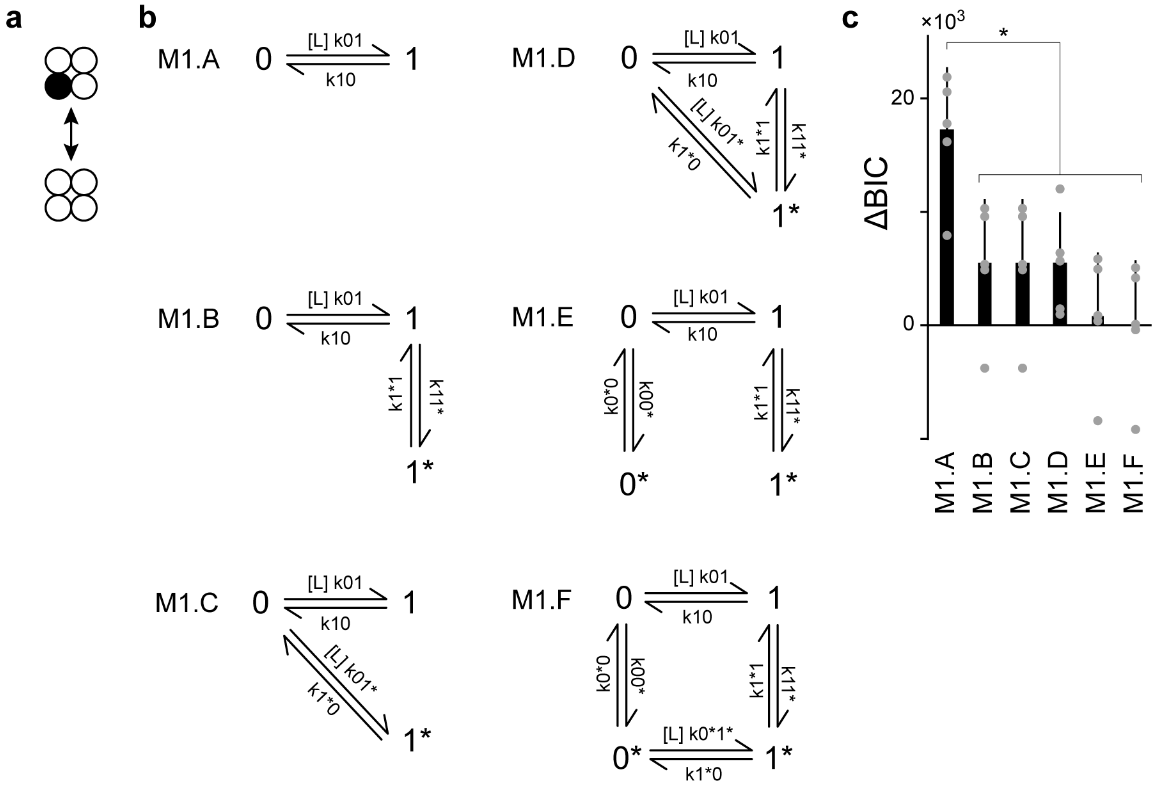

Supplementary Fig. 9 Single site dynamics for the first binding event. a Schematic illustrating binding to the first of four sites per channel. CNBDs depicted in unbound (open circles) and bound (filled circle) conformations. **b** Evaluated single site models describing binding and conformational exchange between distinct bound or unbound states. State names indicate the number of bound ligands and an asterix denotes distinct states having the same number of bound sites. Rate constants are in $\mu M^{-1}s^{-1}$ for binding transitions ($0 \rightarrow 1$, $0 \rightarrow 1^*$, $0^* \rightarrow 1^*$) and s^{-1} for all other transitions. [L] indicates ligand concentration for binding steps. Rate constants were optimized in QuB for data segments comprised of isolated binding events (no stacked events) across all molecules and concentrations (see Methods in main text). For models M1.D and M1.F one of the rate constants was constrained to enforce microscopic reversibility in the loop. Rate constants and their estimated errors are given in Supplementary Table 2. **c** BIC scores for the models shown in C relative to the model with the best score (smaller score is better; $\Delta BIC = BIC - BIC_{best\ model}$). Bars are ΔBIC scores for model fits to the entire dataset. Error bars are standard deviations across BIC scores for five randomized folds of the dataset where each fold contained ~20% of the data for each concentration (data points are BIC scores for each fold mean-centered on their associated bars). Significance was determined by one-way ANOVA with post-hoc Tukey test at $^*p < 0.05$. See source data file for exact p values.

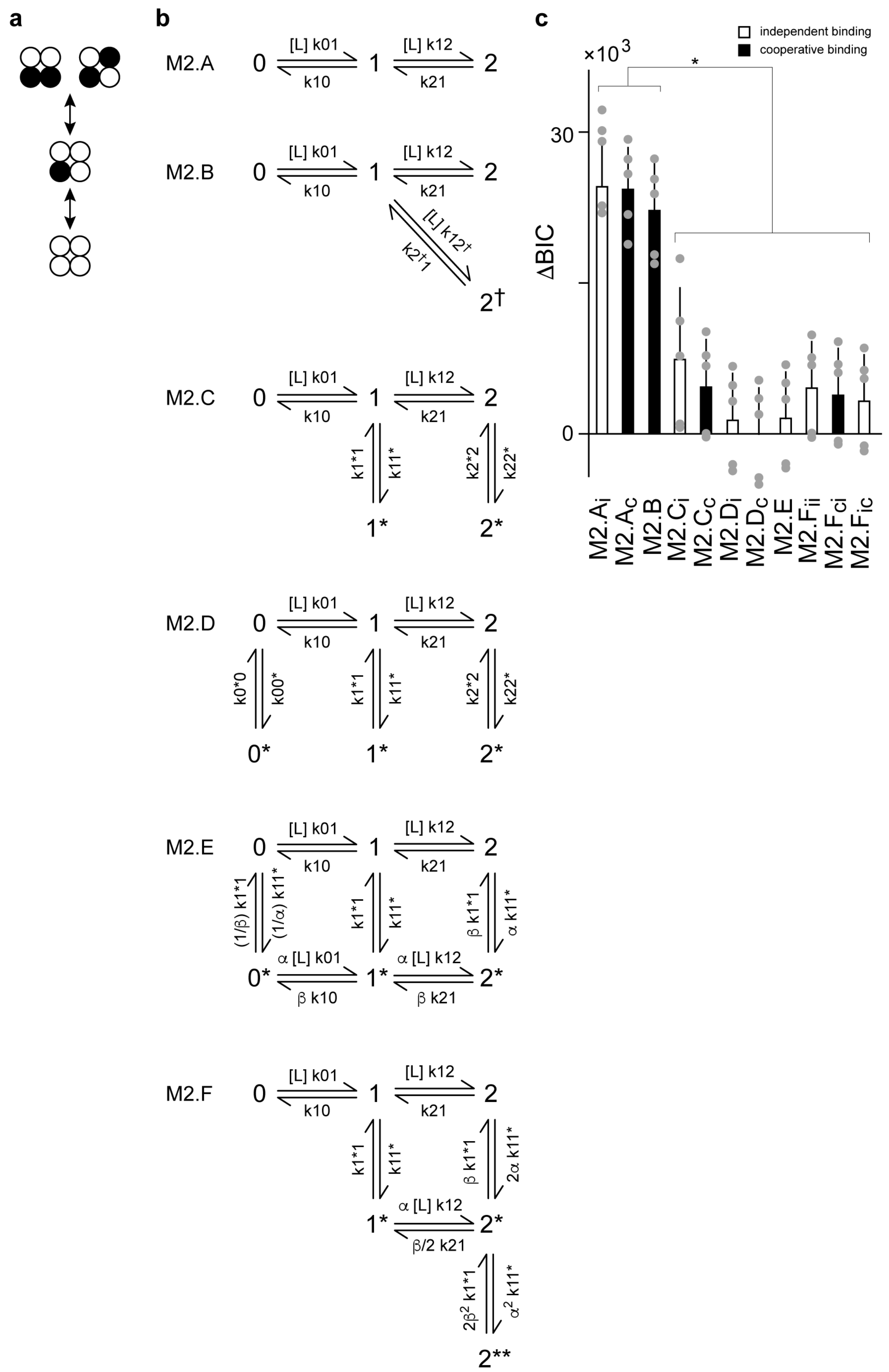

Supplementary Fig. 10 Dynamics for the first two binding events. a Schematic illustrating

binding to the first two of four sites per channel. CNBDs depicted in unbound (open circles) and bound (filled circle) conformations. **b** Evaluated two site models describing binding and conformational exchange between distinct bound or unbound states. State names indicate the number of bound ligands, and an asterisk denotes distinct states having the same number of occupied sites. Rate constants are in $\mu M^{-1}s^{-1}$ for binding transitions ($0 \rightarrow 1$, $1 \rightarrow 2$, $1 \rightarrow 2^{\dagger}$, $0^{*} \rightarrow 1^{*}$, $1^{*} \rightarrow 2^{*}$) and s^{-1} for all other transitions. [L] indicates ligand concentration for binding steps. M2.A is the simplest possible scheme for sequential binding at two sites. M2.B allows for two distinct di-liganded states (e.g., adjacent and diagonally opposed occupied sites as depicted in *a*). M2.C, M2.D and M2.E extend M2.A with a global conformational change of both sites (between states i and i*). M2.F describes binding followed by a conformational change at each site separately. Note that only model M2.B distinguishes between adjacent and diagonal doubly bound conformations in the channel tetramer. Each model is further subdivided into several models sharing the same schematic but differing in the applied constraints. For example, M2.A_i and M2.A_c both have the M2.A structure, but A_i constrains the two binding steps to be identical and independent, whereas A_c allows the dynamics for the two steps to differ (i.e. allows cooperativity between binding sites). Models C_i and C_c (and also D_i and D_c) share the same constraints as A_i and A_c. M2.E is a MWC model for binding and a global conformational exchange of the binding sites. M2.F_{ii} assumes completely independent and identical sites, whereas M2.F_{ci} allows for binding of the 2nd ligand to differ from that of the first (binding cooperativity), and M2.F_{ic} allows the conformational change following binding at either site to depend on the number of bound ligands. See Supplementary Table 3 for a description of all applied constraints. Rate constants were optimized in QuB for the entire data set across all molecules and concentrations excluding events with more than two bound ligands (see Methods in main text). Rate constants and their estimated errors are given in Supplementary Table 3 along with model constraints. **c** BIC scores for each model relative to the model with the best score (smaller score is better; $\Delta BIC = BIC - BIC_{best\ model}$). See description above for model subscripts. Bars are ΔBIC scores for model fits to the entire dataset. Error bars are standard deviations across BIC scores for five randomized folds of the dataset where each fold contained ~20% of the data for each concentration (data points are BIC scores for each fold mean-centered on their associated bars). Significance was determined by one-way ANOVA with post-hoc Tukey test at $*p < 0.05$. See source data file for exact p values.

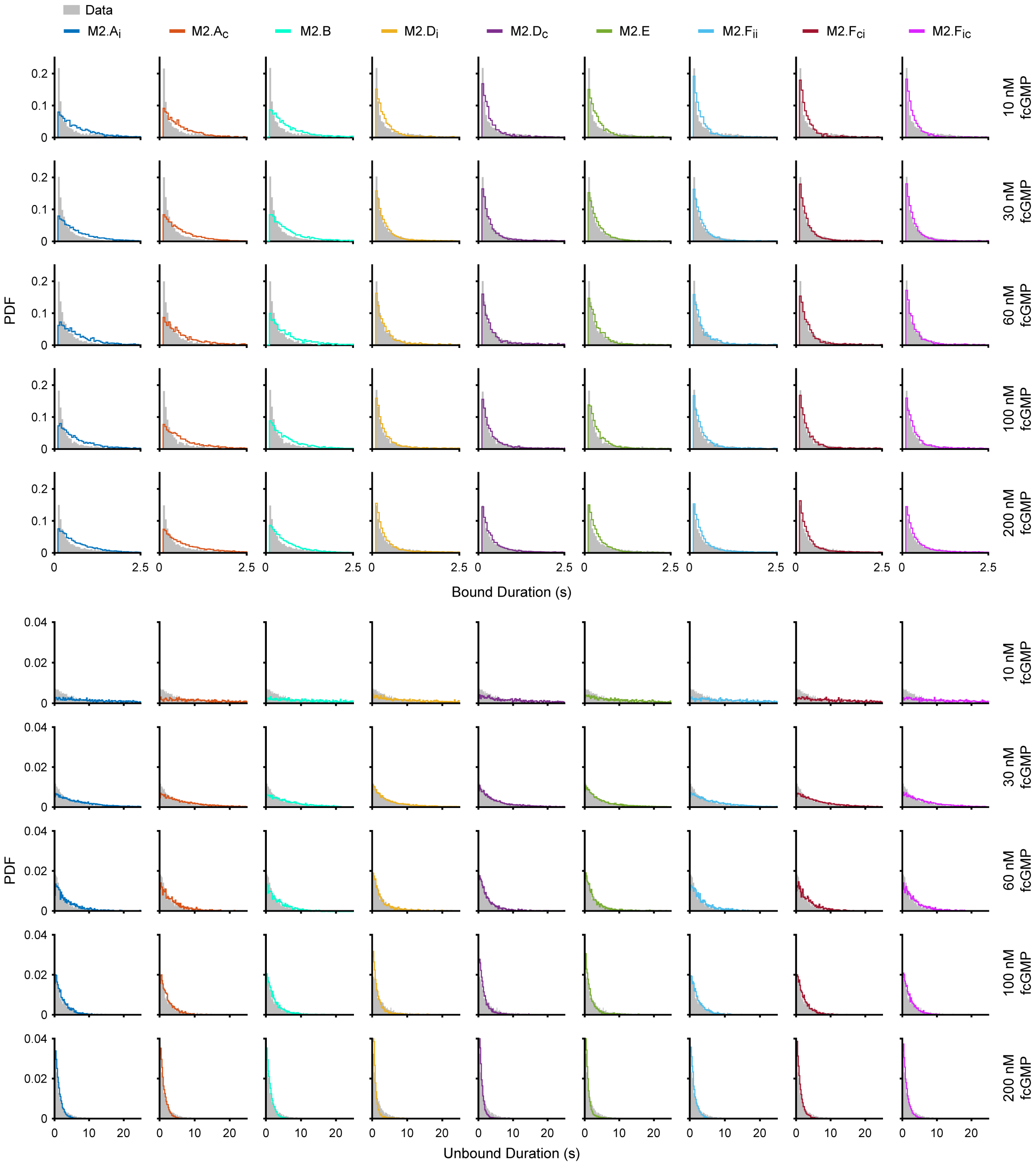

Supplementary Fig. 11 Comparison of observed dwell time distributions with predictions for two-site models. Experimentally observed bound and unbound dwell time distributions (gray) overlaid with predicted distributions from simulated bound time series for each two-site model (colored; see Supplementary Fig. 10). For each ligand concentration, simulations matched the length of data collected.

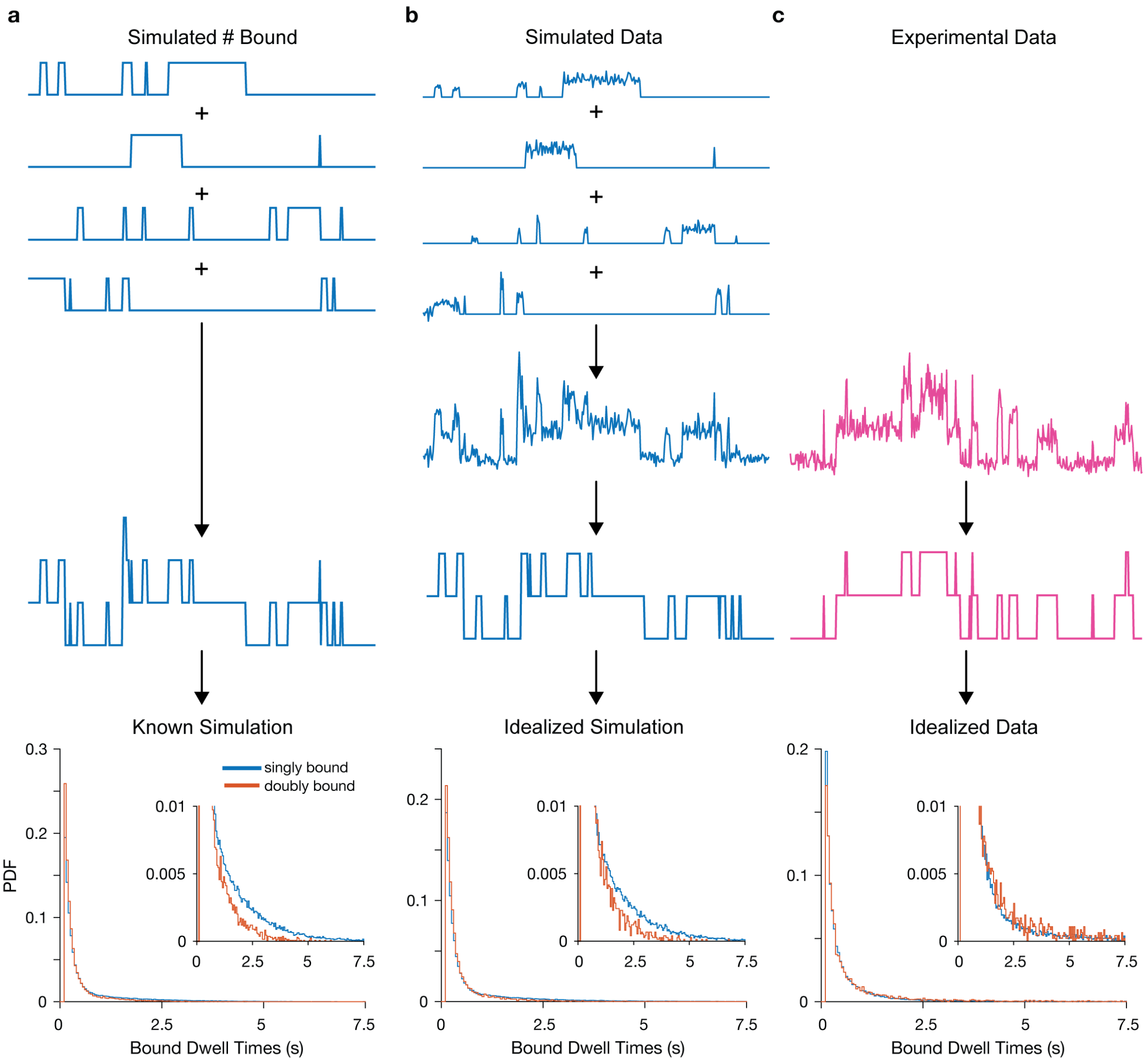

Supplementary Fig. 12 Comparison of experimental data with simulations for independent CNBDs. **a** Four simulated single-site bound time series were added together to generate bound series representative of tetramers comprised of independent CNBDs. **b** Gaussian noise and event amplitude heterogeneity were added to simulated bound series to reflect experimental observations, and the noisy simulations were idealized to obtain bound series. **c** Experimental fcGMP binding fluorescence traces idealized to obtain estimated bound series in exactly the same way as for the simulated data in (b). Dwell time distribution in singly bound (blue; one of four CNBDs occupied) and doubly bound (red; two of four CNBDs occupied) states are shown for both known simulations (a), idealized simulations after adding noise (b), and experimental fcGMP fluorescence traces (c). The reduction in long-lived doubly bound events as compared to singly bound events in the simulated data is a consequence of the fact that unbinding of either ligand will exit a doubly bound state.

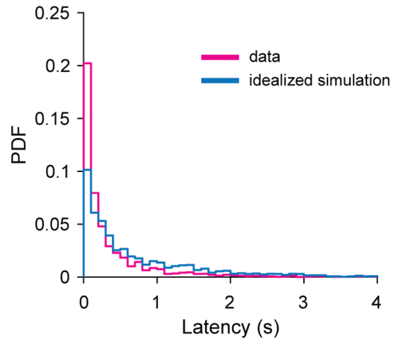

Supplementary Fig. 13 Latency between 1st and 2nd binding events. Distribution of latencies to binding of the second ligand after binding the first ligand. Comparison between our experimental data (magenta) and simulations for four independent sites (blue, see Methods in main text).

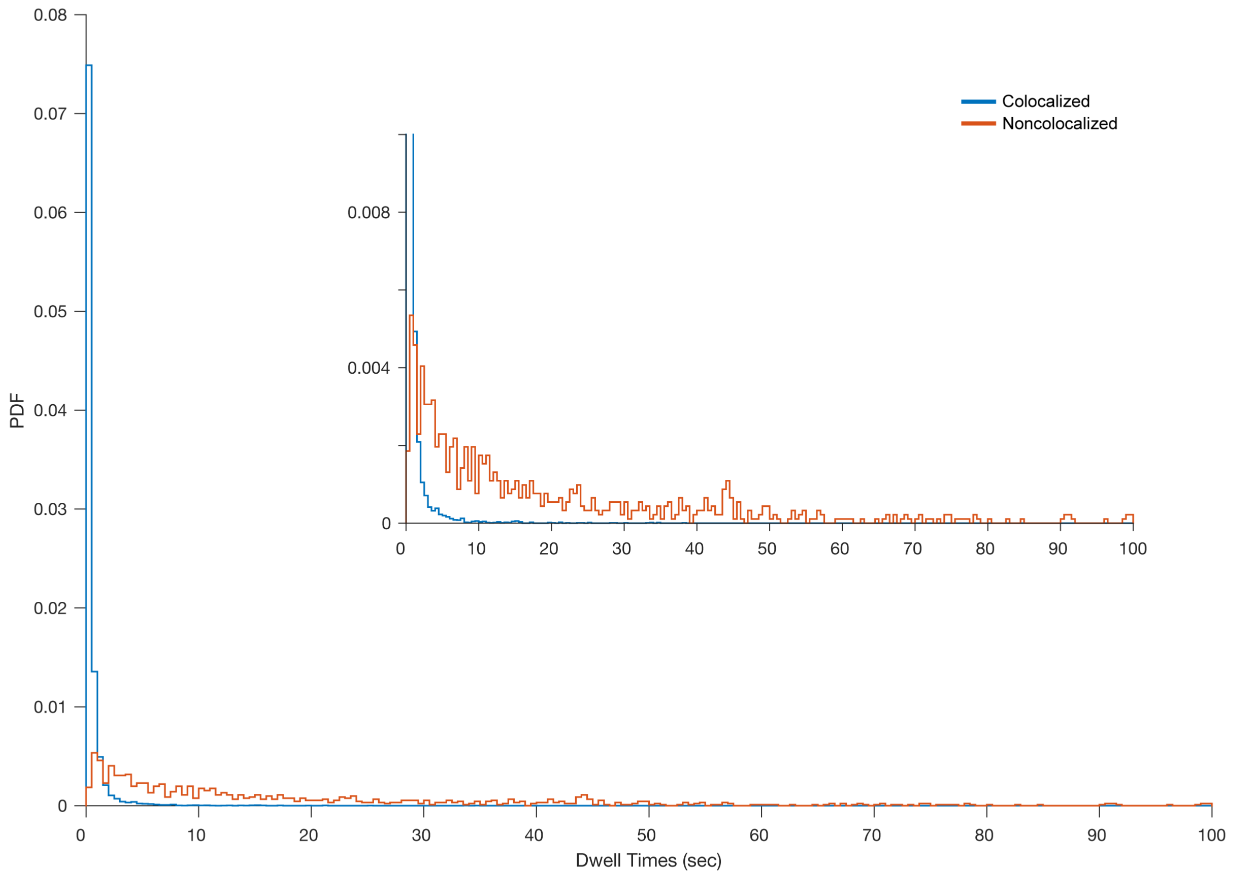

Supplementary Fig. 14 Photobleaching of single fcGMP molecules. Lifetimes of individual fcGMP molecules at noncolocalized spots assumed to largely reflect dye adsorption to the surface and subsequent termination by photobleaching. The abscissa is limited to the first 100 seconds for visualization, although lifetimes up to 143 seconds were observed. Lifetimes of colocalized binding events at TAX-4 channels in 30 nM fcGMP are shown for comparison. The mean lifetime for noncolocalized events was 23.4 seconds, an order of magnitude longer than the time constant for the longest duration bound component (Supplementary Table 1).

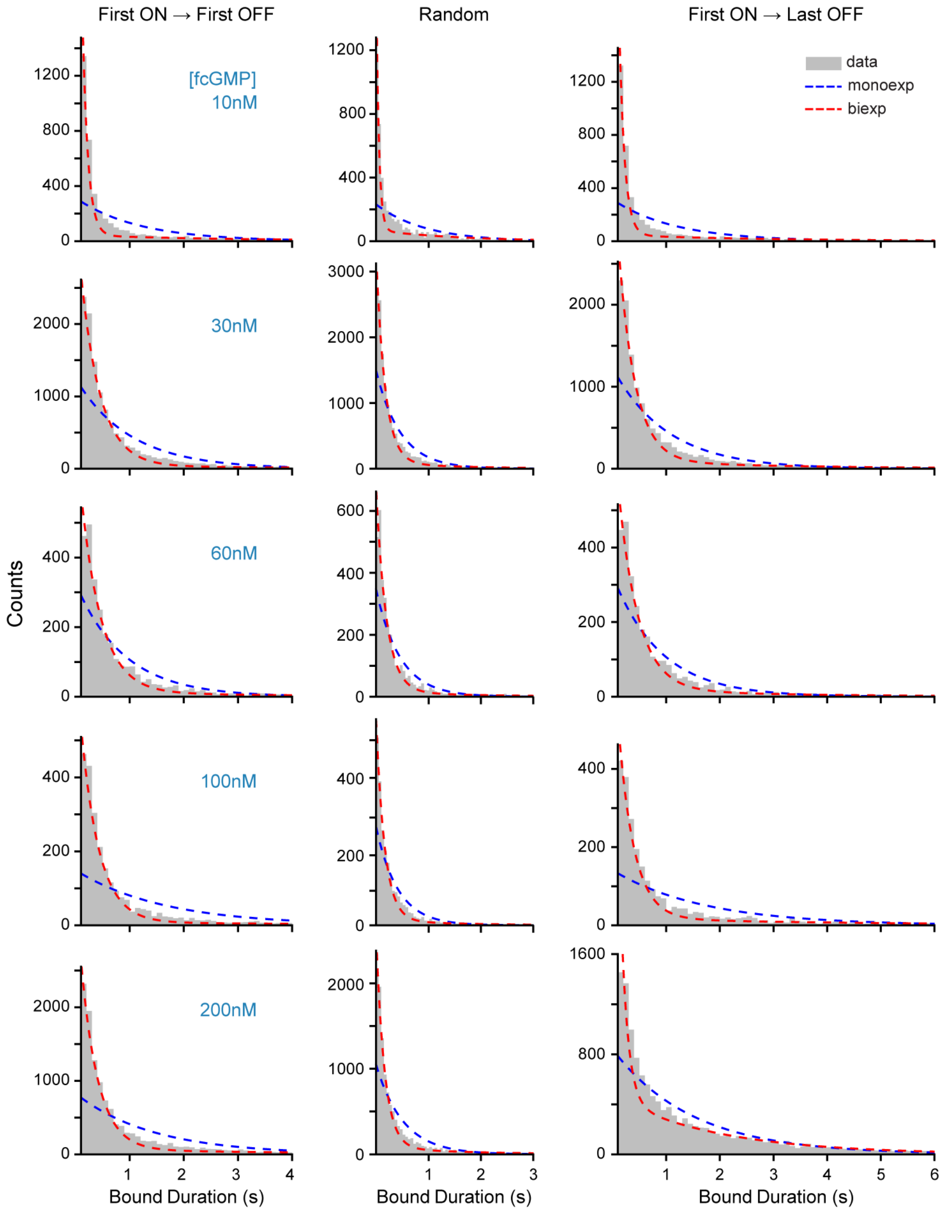

Supplementary Fig. 15 Bound dwell time distributions under the extreme assumptions that the first ligand to bind is always either the first or the last to unbind or where the order of unbinding is determined randomly. Dwell time distributions for data (gray) overlaid with mono- (blue dashed) and bi-exponential (red dashed) maximum likelihood fits. The random assignment distributions are also shown in Fig. 5b. Biexponential time constants (and relative amplitude) for first on → first off: 10 nM: 0.05 s (0.81), 1.47 s (0.19); 30 nM: 0.18 s (0.87), 2.16 s (0.13); 60 nM: 0.19 s (0.86), 1.66 s (0.14); 100 nM: 0.17 s (0.82), 3.10 s (0.18); 200 nM: 0.16 s (0.79), 2.26 s (0.21). Biexponential time constants (and relative amplitude) for first on → last off: 10 nM: 0.05 s (0.80), 1.42 s (0.20); 30 nM: 0.15 s (0.79), 1.51 s (0.21); 60 nM: 0.18 s (0.82), 1.39 s (0.18); 100 nM: 0.15 s (0.72), 2.18 s (0.28); 200 nM: 0.06 s (0.43), 0.98 s (0.57). Biexponential time constants and relative amplitudes for random unbinding order are given in Supplementary Table 1.

[fcGMP] (nM)	Bound Durations τ1 (s) [95% CI]	τ2 (s) [95% CI]	A1 [95% CI]	A2	Unbound Durations τ1 (s) [95% CI]	τ2 (s) [95% CI]	A1 [95% CI]	A2
10	0.05 [0.04-0.06]	1.6 [1.5-1.7]	0.81 [0.78-0.83]	0.19	4.2 [3.9-4.5]	21 [19-23]	0.76 [0.68-0.84]	0.24
30	0.17 [0.16-0.18]	1.8 [1.7-1.9]	0.83 [0.80-0.86]	0.17	3.8 [3.6-4.0]	12 [11-13]	0.75 [0.65-0.85]	0.25
60	0.19 [0.17-0.21]	1.6 [1.4-1.8]	0.85 [0.79-0.91]	0.15	2.1 [1.7-2.5]	11 [10-12]	0.85 [0.83-0.87]	0.15
100	0.15 [0.14-0.16]	2.5 [2.3-2.7]	0.79 [0.75-0.83]	0.21	1.7 [1.5-1.9]	4.1 [2.0-6.2]	0.55 [0.45-0.65]	0.45
200	0.18 [0.17-0.19]	2.4 [2.3-2.5]	0.81 [0.78-0.84]	0.19	1.3 [1.2-1.4]	5.7 [4.9-6.5]	0.82 [0.75-0.89]	0.18

Supplementary Table 1. Time constants (τ) and relative weights (A) for biexponential maximum likelihood fits to bound and unbound dwell time distributions across tested fcGMP concentrations.

Model	$k_{0\to1}$	$k_{1\to0}$	$k_{1\to1*}$	$k_{1*\to1}$	$k_{0\to0*}$	$k_{0*\to0}$	$k_{0*\to1*}$	$k_{1*\to0*}$	$k_{0\to1*}$	$k_{1*\to0}$	ΔBIC
M1.A	4.2 ± 0.2	2.3 ± 0.04	–	–	–	–	–	–	–	–	17175 ± 5555
M1.B	4.4 ± 0.2	4.5 ± 0.1	0.54 ± 0.06	0.54 ± 0.05	–	–	–	–	–	–	5412 ± 5688
M1.C	3.8 ± 0.1	5.1 ± 0.2	–	–	–	–	–	–	0.58 ± 0.07	0.47 ± 0.04	5412 ± 5687
M1.D	4.4 ± 0.2	4.5 ± 0.1	0.54 ± 0.06	0.54 ± 0.05	–	–	–	–	5.7e-8 ± 0.1e-8	5.8e-7 ± 0.1e-7	5427 ± 4544
M1.E	7.0 ± 0.3	4.5 ± 0.1	0.54 ± 0.06	0.54 ± 0.05	0.06 ± 0.003	0.17 ± 0.01	–	–	–	–	797 ± 5736
M1.F	7.4 ± 0.3	4.7 ± 0.1	0.25 ± 0.04	0.27 ± 0.03	0.03 ± 0.001	0.05 ± 0.002	0.74 ± 0.37	0.31 ± 0.02	–	–	0 ± 5737

Supplementary Table 2. Rate constants and relative BIC scores for single site models (Supplementary Fig. 9). Units are $\mu M^{-1}s^{-1}$ for binding transitions ($k_{0\to1}$, $k_{0\to1*}$, $k_{0*\to1*}$) and s^{-1} for all other transitions. Rate constants were optimized in QuB for data segments comprised of isolated binding events (no stacked events) across all molecules and concentrations (see Methods in main text). For models M1.D and M1.F one of the rate constants was constrained to enforce microscopic reversibility in the loop. Errors are standard deviations across optimized rate constants or BIC scores for five randomized folds of the dataset where each fold contained ~20% of the data for each concentration.

Model	$k_{0\to1}$	$k_{1\to0}$	$k_{1\to2}$	$k_{2\to1}$	$k_{1\to2^\dagger}$	$k_{2^\dagger\to1}$	$k_{0\to0^*}$	$k_{0^*\to0}$	$k_{1\to1^*}$	$k_{1^*\to1}$	$k_{2\to2^*}$	$k_{2^*\to2}$	α	β	ΔBIC
M2.A$_i$	4.4 ± 0.1	1.6 ± 0.04	$\frac{3}{4}k_{0\to1}$	$2\,k_{1\to0}$	–	–	–	–	–	–	–	–	–	–	27209 ± 4655
M2.A$_c$	4.4 ± 0.1	1.8 ± 0.1	3.4 ± 0.1	1.6 ± 0.1	–	–	–	–	–	–	–	–	–	–	24584 ± 4278
M2.B	4.4 ± 0.1	1.9 ± 0.1	3.0 ± 0.1	4.2 ± 0.2	0.50 ± 0.03	0.30 ± 0.02	–	–	–	–	–	–	–	–	22191 ± 4689
M2.C$_i$	5.0 ± 0.1	3.5 ± 0.1	$\frac{3}{4}k_{0\to1}$	$2\,k_{1\to0}$	–	–	–	–	0.42 ± 0.03	0.49 ± 0.02	2.0 ± 0.1	0.80 ± 0.02	–	–	7397 ± 7182
M2.C$_c$	4.7 ± 0.1	4.2 ± 0.1	8.5 ± 0.3	3.6 ± 0.1	–	–	–	–	0.77 ± 0.05	0.68 ± 0.03	2.1 ± 0.03	0.84 ± 0.03	–	–	4640 ± 4804
M2.D$_i$	8.0 ± 0.2	3.6 ± 0.1	$\frac{3}{4}k_{0\to1}$	$2\,k_{1\to0}$	–	–	0.11 ± 0.02	0.24 ± 0.03	0.51 ± 0.04	0.55 ± 0.03	2.1 ± 0.1	0.84 ± 0.02	–	–	1338 ± 4757
M2.D$_c$	7.0 ± 0.1	4.2 ± 0.1	8.5 ± 0.3	3.7 ± 0.1	–	–	0.06 ± 0.01	0.18 ± 0.02	0.77 ± 0.05	0.68 ± 0.03	0.50 ± 0.03	0.40 ± 0.03	–	–	0 ± 4748
M2.E	8.0 ± 0.2	3.8 ± 0.1	$\frac{3}{4}k_{0\to1}$	$2\,k_{1\to0}$	–	–	$\frac{1}{\alpha}\,k_{1\to1*}$	$\frac{1}{\beta}\,k_{1*\to1}$	0.01 ±0.001	0.01 ±0.001	$\alpha\,k_{1\to1*}$	$\beta\,k_{1*\to1}$	0.18 ±0.005	0.11 ±0.003	1537 ± 4764
M2.F$_{ii}$	4.6 ± 0.1	3.9 ± 0.1	$\frac{3}{4}k_{0\to1}$	$2\,k_{1\to0}$	–	–	–	–	0.46 ± 0.04	0.34 ± 0.02	$2\alpha\,k_{1\to1*}$	$\beta\,k_{1*\to1}$	1	1	4550 ± 4687
M2.F$_{ic}$	4.8 ± 0.1	4.1 ± 0.1	$\frac{3}{4}k_{0\to1}$	$2\,k_{1\to0}$	–	–	–	–	0.53 ± 0.04	0.44 ± 0.02	$2\alpha\,k_{1\to1*}$	$\beta\,k_{1*\to1}$	0.75 ± 0.04	0.49 ± 0.02	3834 ± 4712
M2.F$_{ci}$	4.7 ± 0.1	4.3 ± 0.1	3.5 ± 0.1	4.1 ± 0.2	–	–	–	–	0.44 ± 0.04	0.34 ± 0.02	$2\alpha\,k_{1\to1*}$	$\beta\,k_{1*\to1}$	1	1	3236 ± 4697

Supplementary Table 3. Rate constants and relative BIC scores for models of the first two binding steps (Supplementary Fig. 10). Units are $\mu M^{-1}s^{-1}$ for binding transitions ($k_{0\to1}$, $k_{1\to2}$, $k_{0*\to1*}$, $k_{1*\to2*}$, $k_{1\to2^\dagger}$) and s^{-1} for all other transitions. Gray shaded cells indicate constraints, and all other cells were free parameters. All constraints for $k_{1\to2}$ and $k_{2\to1}$ reflect the statistical factors associated with independent binding at each site in a tetramer, whereas otherwise the binding steps were allowed to differ (i.e., be cooperative). Constraints for model M2.E transitions $k_{i\to i*}$ and $k_{i*\to i}$ reflect the additive effect of ligand binding on the conformational change between states i and i* as depicted by the factors α and β in Supplementary Fig. 10. For all three M2.F models, constraints on transitions not involving ligand binding/unbinding imply that the ratio of the transition rate for the conformational change in doubly liganded states (2 → 2*) to that in singly liganded states (1 → 1*) is given by α, and the factor of 2 is a statistical factor given that either site could undergo the conformational change. Likewise, the ratio of the reverse transition rates for 2* → 2 and 1* → 1 is given by β. Constraints for all M2.F models not shown in the table are $k_{1*\to2*} = \alpha\,k_{1\to2}$, $k_{2*\to1*} = (\beta/2)\;k_{2\to1}$, $k_{2*\to2**} = (\alpha/2)\,k_{2\to2*}$ and $k_{2**\to2*} = 2\beta\;k_{2*\to2}$. Rate constants were optimized in QuB for the entire data set across all molecules and concentrations excluding events with

more than two bound ligands (see Methods in main text). Errors are standard deviations across optimized rate constants or BIC scores for five randomized folds of the dataset where each fold contained ~20% of the data for each concentration.

Model	$\boldsymbol{K_{0\to1}}$	$\boldsymbol{K_{1\to2}}$	$\boldsymbol{K_{1\to2}}/\left(\frac{3}{8}K_{0\to1}\right)$	$\boldsymbol{K_{1\to2^\dagger}}$	$\boldsymbol{K_{1\to2^\dagger}}/\left(\frac{3}{8}K_{0\to1}\right)$	$\boldsymbol{K_{0\to0^*}}$	$\boldsymbol{K_{1\to1^*}}$	$\boldsymbol{K_{2\to2^*}}$	α/β
M2.A_i	2.8	$\frac{3}{8}K_{0\to1}$	1	–	–	–	–	–	–
M2.A_c	2.4	2.1	2.3	–	–	–	–	–	–
M2.B	2.3	0.71	0.82	1.7	2.0	–	–	–	–
M2.C_i	1.4	$\frac{3}{8}K_{0\to1}$	1	–	–	–	0.86	2.5	–
M2.C_c	1.1	2.4	5.8	–	–	–	1.1	2.5	–
M2.D_i	2.2	$\frac{3}{8}K_{0\to1}$	1	–	–	0.46	0.93	2.5	–
M2.D_c	1.7	2.3	3.6	–	–	0.33	1.1	1.3	–
M2.E	2.1	$\frac{3}{8}K_{0\to1}$	1	–	–	$\frac{\beta}{\alpha}K_{1\to1*}$	1.0	$\frac{\alpha}{\beta}K_{1\to1*}$	1.6
M2.F_{ii}	1.2	$\frac{3}{8}K_{0\to1}$	1	–	–	–	1.4	$\frac{2\alpha}{\beta}K_{1\to1*}$	1
M2.F_{ic}	1.2	$\frac{3}{8}K_{0\to1}$	1	–	–	–	1.2	$2\alpha\, k_{1\to1*}$	1.5
M2.F_{ci}	1.1	0.85	2.1	–	–	–	1.3	$2\alpha\, k_{1\to1*}$	1

Supplementary Table 4. Equilibrium constants (e.g., $\boldsymbol{K_{0\to1}} = k_{0\to1}/k_{1\to0}$) for models of the first two binding steps (Supplementary Fig. 10; Supplementary Table 3). Units are μM for binding/unbinding steps and unitless for all other pairs of transitions. Gray shaded cells indicate constraints, and all other cells were free parameters (see Supplementary Table 3). Statistical factors of 3/8 indicate binding to independent and identical sites. Ratios of equilibrium constants for the second binding step relative to that for independent and identical sites indicate positive or negative binding cooperativity (i.e., greater or less than one, respectively).

Model	$k_{0\to1}$	$k_{1\to0}$	$k_{1\to2}$	$k_{2\to1}$	$k_{0\to0^*}$	$k_{0^*\to0}$	$k_{1\to1^*}$	$k_{1^*\to1}$	$k_{2\to2^*}$	$k_{2^*\to2}$	α	β	ΔBIC
M2.C_{p10}	4.6	4.6	$\sqrt{10}\times\frac{3}{4}k_{0\to1}$	$\frac{1}{\sqrt{10}}\times 2\,k_{1\to0}$	–	–	0.99	0.77	0.37	0.4	–	–	4958
M2.C_{n10}	5.1	2.8	$\frac{1}{\sqrt{10}}\times\frac{3}{4}k_{0\to1}$	$\sqrt{10}\times 2\,k_{1\to0}$	–	–	0.24	0.37	5.0	0.4	–	–	18860
M2.C_{p100}	4.2	8.1	$10\times\frac{3}{4}k_{0\to1}$	$\frac{1}{10}\times 2\,k_{1\to0}$	–	–	4.1	1.5	0.15	0.4	–	–	13240
M2.C_{n100}	5.3	3.7	$\frac{1}{10}\times\frac{3}{4}k_{0\to1}$	$10\times 2\,k_{1\to0}$	–	–	0.34	0.45	57	0.4	–	–	31904
M2.D_{p10}	6.1	4.9	$\sqrt{10}\times\frac{3}{4}k_{0\to1}$	$\frac{1}{\sqrt{10}}\times 2\,k_{1\to0}$	0.04	0.18	1.2	0.85	0.39	0.4	–	–	1145
M2.D_{n10}	9.1	2.9	$\frac{1}{\sqrt{10}}\times\frac{3}{4}k_{0\to1}$	$\sqrt{10}\times 2\,k_{1\to0}$	0.11	0.18	0.26	0.39	5.1	0.4	–	–	11187
M2.D_{p100}	5.3	9.6	$10\times\frac{3}{4}k_{0\to1}$	$\frac{1}{10}\times 2\,k_{1\to0}$	0.02	0.18	6.2	1.9	0.18	0.4	–	–	11081
M2.D_{n100}	1.0	3.4	$\frac{1}{10}\times\frac{3}{4}k_{0\to1}$	$10\times 2\,k_{1\to0}$	0.13	0.18	0.35	0.46	57	0.4	–	–	23519
M2.E_{p10}	5.2	4.7	$\frac{3}{4}k_{0\to1}$	$2\,k_{1\to0}$	$\frac{1}{\alpha}\,k_{1\to1*}$	$\frac{1}{\beta}\,k_{1*\to1}$	0.90	0.75	$\alpha\,k_{1\to1*}$	$\beta\,k_{1*\to1}$	$\sqrt{10}$	$\frac{1}{\sqrt{10}}$	6161
M2.E_{n10}	8.0	3.7	$\frac{3}{4}k_{0\to1}$	$2\,k_{1\to0}$	$\frac{1}{\alpha}\,k_{1\to1*}$	$\frac{1}{\beta}\,k_{1*\to1}$	0.02	0.01	$\alpha\,k_{1\to1*}$	$\beta\,k_{1*\to1}$	$\frac{1}{\sqrt{10}}$	10	13012
M2.E_{p100}	5.7	6.4	$\frac{3}{4}k_{0\to1}$	$2\,k_{1\to0}$	$\frac{1}{\alpha}\,k_{1\to1*}$	$\frac{1}{\beta}\,k_{1*\to1}$	1.7	0.76	$\alpha\,k_{1\to1*}$	$\beta\,k_{1*\to1}$	10	$\frac{1}{10}$	13867
M2.E_{n100}	53	5.9	$\frac{3}{4}k_{0\to1}$	$2\,k_{1\to0}$	$\frac{1}{\alpha}\,k_{1\to1*}$	$\frac{1}{\beta}\,k_{1*\to1}$	0.23	0.03	$\alpha\,k_{1\to1*}$	$\beta\,k_{1*\to1}$	$\frac{1}{10}$	10	32375
M2.F_{p10}	4.5	4.9	$\sqrt{10}\times\frac{3}{4}k_{0\to1}$	$\frac{1}{\sqrt{10}}\times 2\,k_{1\to0}$	–	–	0.78	0.52	$2\alpha\,k_{1\to1*}$	$\beta\,k_{1*\to1}$	1	1	3951
M2.F_{n10}	4.9	4.7	$\frac{1}{\sqrt{10}}\times\frac{3}{4}k_{0\to1}$	$\sqrt{10}\times 2\,k_{1\to0}$	–	–	1.1	0.73	$2\alpha\,k_{1\to1*}$	$\beta\,k_{1*\to1}$	1	1	12415
M2.F_{p100}	4.2	8.0	$10\times\frac{3}{4}k_{0\to1}$	$\frac{1}{10}\times 2\,k_{1\to0}$	–	–	4.0	1.0	$2\alpha\,k_{1\to1*}$	$\beta\,k_{1*\to1}$	1	1	13259
M2.F_{n100}	4.9	5.8	$\frac{1}{10}\times\frac{3}{4}k_{0\to1}$	$10\times 2\,k_{1\to0}$	–	–	1.8	0.97	$2\alpha\,k_{1\to1*}$	$\beta\,k_{1*\to1}$	1	1	15375

Supplementary Table 5. Rate constants and relative BIC scores for models of the first two binding steps (Supplementary Fig. 10;

Supplementary Table 3) where either positive or negative cooperativity between sites was enforced. Units are $\mu M^{-1}s^{-1}$ for binding transitions ($k_{0\rightarrow1}$, $k_{1\rightarrow2}$) and s^{-1} for all other transitions. Gray shaded cells indicate constraints, and all other cells were free parameters. For models M2.C, M2.D and M2.F, positive or negative cooperativity was enforced for the second binding step relative to the first. For positive cooperativity $k_{1\rightarrow2} = fk_{0\rightarrow1}$ and $k_{2\rightarrow1} = (1/f)k_{1\rightarrow0}$, where f was either $\sqrt{10}$ or 10. This results in a 10- or 100-fold increase in the equilibrium constant for the second binding step relative to the first. For negative cooperativity $k_{1\rightarrow2} = (1/f)k_{0\rightarrow1}$ and $k_{2\rightarrow1} = fk_{1\rightarrow0}$, where f was either $\sqrt{10}$ or 10. This results in a 10- or 100-fold decrease in the equilibrium constant for the second binding step relative to the first. For model M2.E, binding and unbinding from each site was considered to be identical and independent, and positive or negative cooperativity was enforced for the factor by which ligand binding influences the global conformational exchange. For positive cooperativity $\alpha = f$ and $\beta = (1/f)$, where f was either $\sqrt{10}$ or 10. This results in a 10- or 100-fold increase in the equilibrium constant for the conformational exchange upon binding of each ligand. For negative cooperativity $\alpha = (1/f)$ and $\beta = f$ where f was either $\sqrt{10}$ or 10. This results in a 10- or 100-fold decrease in the equilibrium constant for the conformational exchange upon binding of each ligand. Constraints for all M2.F models not shown in the table are $k_{1*\rightarrow2*} = \alpha\ k_{1\rightarrow2}$, $k_{2*\rightarrow1*} = (\beta/2)\ k_{2\rightarrow1}$, $k_{2*\rightarrow2**} = (\alpha/2)\ k_{2\rightarrow2*}$ and $k_{2**\rightarrow2*} = 2\beta\ k_{2*\rightarrow2}$. Rate constants were optimized in QuB for the entire data set across all molecules and concentrations excluding events with more than two bound ligands (see Methods in main text).

References

1. Fu, X. *et al.* High-throughput fluorescence correlation spectroscopy enables analysis of surface components of cell-derived vesicles. *Anal. Bioanal. Chem.* **412**, 2589–2597 (2020).
2. Fox-Loe, A. M., Moonschi, F. H. & Richards, C. I. Organelle-specific single-molecule imaging of α4β2 nicotinic receptors reveals the effect of nicotine on receptor assembly and cell-surface trafficking. *J. Biol. Chem.* **292**, 21159–21169 (2017).
