# Supplementary material for: Single-molecule imaging with cell-derived nanovesicles reveals early binding dynamics at a cyclic nucleotide-gated ion channel
Source: Nat Commun. 2021 Nov 9;12:6459. doi: 10.1038/s41467-021-26816-5 (PMC8578382; doi:10.1038/s41467-021-26816-5)
Supplement: Supplementary file 3 — Description of Additional Supplementary Files [file 41467_2021_26816_MOESM3_ESM.pdf]

Description of Additional Supplementary Files

File Name: Supplementary Software 1
Description: Experimental data for single-molecule fcGMP binding time series, simulated binding time series and analysis scripts as MATLAB files.
