# Supplementary material for: Single-molecule imaging with cell-derived nanovesicles reveals early binding dynamics at a cyclic nucleotide-gated ion channel
Source: Nat Commun. 2021 Nov 9;12:6459. doi: 10.1038/s41467-021-26816-5 (PMC8578382; doi:10.1038/s41467-021-26816-5)
Supplement: Supplementary file 5 — Reporting Summary [file 41467_2021_26816_MOESM5_ESM.pdf]

nature research

Corresponding author(s): Marcel P. Goldschen-Ohm

Last updated by author(s): Oct 18, 2021

Reporting Summary

Nature Research wishes to improve the reproducibility of the work that we publish. This form provides structure for consistency and transparency in reporting. For further information on Nature Research policies, see our Editorial Policies and the Editorial Policy Checklist.

Statistics

For all statistical analyses, confirm that the following items are present in the figure legend, table legend, main text, or Methods section.

n/a	Confirmed	
☐	☒	The exact sample size (*n*) for each experimental group/condition, given as a discrete number and unit of measurement
☐	☒	A statement on whether measurements were taken from distinct samples or whether the same sample was measured repeatedly
☐	☒	The statistical test(s) used AND whether they are one- or two-sided<br>*Only common tests should be described solely by name; describe more complex techniques in the Methods section.*
☒	☐	A description of all covariates tested
☒	☐	A description of any assumptions or corrections, such as tests of normality and adjustment for multiple comparisons
☐	☒	A full description of the statistical parameters including central tendency (e.g. means) or other basic estimates (e.g. regression coefficient) AND variation (e.g. standard deviation) or associated estimates of uncertainty (e.g. confidence intervals)
☐	☒	For null hypothesis testing, the test statistic (e.g. *F*, *t*, *r*) with confidence intervals, effect sizes, degrees of freedom and *P* value noted<br>*Give P values as exact values whenever suitable.*
☒	☐	For Bayesian analysis, information on the choice of priors and Markov chain Monte Carlo settings
☒	☐	For hierarchical and complex designs, identification of the appropriate level for tests and full reporting of outcomes
☒	☐	Estimates of effect sizes (e.g. Cohen's *d*, Pearson's *r*), indicating how they were calculated

Our web collection on statistics for biologists contains articles on many of the points above.

Software and code

Policy information about availability of computer code

Data collection	Micro Manager 1.4 for image acquisition, Mad City Labs TIRF Lock for automated focusing, Coherent Connection v3 for laser control
Data analysis	Data analysis was performed using QuB v2.0 and custom-written scripts in MATLAB 2020a available as supplementary material and at https://github.com/marcel-goldschen-ohm/single-molecule-imaging-toolbox.

For manuscripts utilizing custom algorithms or software that are central to the research but not yet described in published literature, software must be made available to editors and reviewers. We strongly encourage code deposition in a community repository (e.g. GitHub). See the Nature Research guidelines for submitting code & software for further information.

Data

Policy information about availability of data

All manuscripts must include a data availability statement. This statement should provide the following information, where applicable:

- Accession codes, unique identifiers, or web links for publicly available datasets
- A list of figures that have associated raw data
- A description of any restrictions on data availability

Source data are provided with this paper. Additional data such as original image series are available from the authors upon request as these files are both large and only a small fraction of their information pertains to the analyzed molecular time series whose data is supplied.

Field-specific reporting

Please select the one below that is the best fit for your research. If you are not sure, read the appropriate sections before making your selection.

- [x] Life sciences
- [ ] Behavioural & social sciences
- [ ] Ecological, evolutionary & environmental sciences

For a reference copy of the document with all sections, see nature.com/documents/nr-reporting-summary-flat.pdf

Life sciences study design

All studies must disclose on these points even when the disclosure is negative.

Sample size	Sample sizes were limited by practicality and throughput and are consistent with previously published similar work in the field. The number of molecules at each concentration was: 10 nM: 112, 30 nM: 325, 60 nM: 63, 100 nM: 65 and 200 nM: 132. In total, we recorded ~60 hours of single-molecule binding.
Data exclusions	Non-colocalized spots and spots with greater than 4 observed bleach steps were excluded as individual molecules are expected to be tetrameric and cannot contain more than 4 bleach steps.
Replication	All single-molecule fluorescent ligand binding experiments were repeated 3-7 times at each tested concentration. Recordings span numerous experiment days for a total of ~60 hours of single-molecule binding across hundreds of molecules. Results were repeatable in all conditions.
Randomization	Data were grouped according to ligand concentration.
Blinding	No blinding was performed for this study, results relied on a clear dependence of activity on ligand concentration.

Reporting for specific materials, systems and methods

We require information from authors about some types of materials, experimental systems and methods used in many studies. Here, indicate whether each material, system or method listed is relevant to your study. If you are not sure if a list item applies to your research, read the appropriate section before selecting a response.

Materials & experimental systems

n/a	Involved in the study	
☐	☒	Antibodies
☐	☒	Eukaryotic cell lines
☒	☐	Palaeontology and archaeology
☒	☐	Animals and other organisms
☒	☐	Human research participants
☒	☐	Clinical data
☒	☐	Dual use research of concern

Methods

n/a	Involved in the study	
☒	☐	ChIP-seq
☒	☐	Flow cytometry
☒	☐	MRI-based neuroimaging

Antibodies

Antibodies used	anti-GFP VHH, conjugated to biotin (ChromoTek, product code: gtb-250).
Validation	ChromoTek reports that this antibody fulfills the highest requirements for antibody validation. We further validated antibody specificity using our nanovesicle pull down approach (see Supplementary Fig. 3).

Eukaryotic cell lines

Policy information about cell lines

Cell line source(s)	ATCC (HEK-293T). Note: cells were disrupted using cavitation for nanovesicle experiments.
Authentication	Cell line was not authenticated.
Mycoplasma contamination	Cell line was not tested for mycoplasma contamination
Commonly misidentified lines (See ICLAC register)	No commonly misidentified cell lines were used in the study.
